# Tunable Mechanical and Electrical Properties of Coaxial Electrospun Composite Nanofibers of P(VDF-TrFE) and P(VDF-TrFE-CTFE)

**DOI:** 10.3390/ijms22094639

**Published:** 2021-04-28

**Authors:** Tu-Ngoc Lam, Chia-Yin Ma, Po-Han Hsiao, Wen-Ching Ko, Yi-Jen Huang, Soo-Yeol Lee, Jayant Jain, E-Wen Huang

**Affiliations:** 1 Department of Materials Science and Engineering, National Yang Ming Chiao Tung University, Hsinchu 30013, Taiwan; lamtungoc1310@gmail.com (T.-N.L.); caroline626936@gmail.com (C.-Y.M.); a0973111453@gmail.com (P.-H.H.); 2 Department of Physics, College of Education, Can Tho University, Can Tho City 900000, Vietnam; 3 Central Region Campus, Industrial Technology Research Institute, Nantou County 54041, Taiwan; wcko@itri.org.tw; 4 Department of Fiber and Composite Materials, Feng Chia University, Taichung 40724, Taiwan; yijenhua@gmail.com; 5 Department of Materials Science and Engineering, Chungnam National University, Daejeon 34134, Korea; sylee2012@cnu.ac.kr; 6 Department of Materials Science and Engineering, Indian Institute of Technology, New Delhi 110016, India; Jayant.Jain@mse.iitd.ac.in

**Keywords:** coaxial electrospun core/shell nanofibers, tensile modulus, wide-angle X-ray diffraction, dielectric constant, piezoelectricity

## Abstract

The coaxial core/shell composite electrospun nanofibers consisting of relaxor ferroelectric P(VDF-TrFE-CTFE) and ferroelectric P(VDF-TrFE) polymers are successfully tailored towards superior structural, mechanical, and electrical properties over the individual polymers. The core/shell-TrFE/CTFE membrane discloses a more prominent mechanical anisotropy between the revolving direction (RD) and cross direction (CD) associated with a higher tensile modulus of 26.9 MPa and good strength-ductility balance, beneficial from a better degree of nanofiber alignment, the increased density, and C-F bonding. The interfacial coupling between the terpolymer P(VDF-TrFE-CTFE) and copolymer P(VDF-TrFE) is responsible for comparable full-frequency dielectric responses between the core/shell-TrFE/CTFE and pristine terpolymer. Moreover, an impressive piezoelectric coefficient up to 50.5 pm/V is achieved in the core/shell-TrFE/CTFE composite structure. Our findings corroborate the promising approach of coaxial electrospinning in efficiently tuning mechanical and electrical performances of the electrospun core/shell composite nanofiber membranes-based electroactive polymers (EAPs) actuators as artificial muscle implants.

## 1. Introduction

Electroactive polymers (EAPs) which change shape or size under applying an electrical stimulus are widely used for diverse applications of electromechanical devices in transducers, sensors, actuators, artificial muscles, smart skin, and soft robotics [1,2,3,4,5,6,7]. The ferroelectric (FE) copolymer poly(vinylidene fluoride-trifluoroethylene) [P(VDF-TrFE)] owning superior piezoelectric properties of highly electroactive polar β phase crystalline structure coupled with a large crystalline domain size discloses striking mechanical properties, high dielectric constant, low dielectric loss, and high electromechanical response in conversion between electrical and mechanical energy [6,8,9,10,11,12]. However, the normal FE polymers generally exhibit lower energy density, broader hysteresis loop, and larger remnant polarization compared with relaxor ferroelectric (RFE) polymers [10,13]. In addition, terpolymers usually have higher dielectric constants than copolymers [10]. As a result, it is highly desirable to achieve a higher dielectric constant and thus higher energy density in P(VDF-TrFE)-based RFE terpolymers rather than in the FE copolymer P(VDF-TrFE). Converting a normal FE into a RFE behavior can be effectively achieved via the physical pinning effect [14]. Introducing the third bulky monomer chlorotrifluoroethylene (CTFE) considered as structural defects into the FE copolymer P(VDF-TrFE) possibly modifies the polar crystalline domain size and molecular conformation change. The resulting RFE terpolymer poly(vinylidene fluoride-trifluoroethylene-chlorotrifluoroethylene) [P(VDF-TrFE-CTFE)] has been reported to possess a narrow hysteresis loop, higher polarization, higher dielectric constant, higher electrostriction, and higher electromechanical response [15,16,17]. An enhancement in the polarization and dielectric responses was attained by blending the RFE terpolymer P(VDF-TrFE-CTFE) with the FE copolymer P(VDF-TrFE) [9,10]. Although comprehensive research has been devoted to elucidating the electroactive properties associated with dielectric and electromechanical responses [9,10], few studies focused on the mechanical properties of the composite sheets consisting of terpolymer and copolymer were extensively examined.

Among the various methods in producing the incorporation of different polymers, electrospinning has recently emerged as one of the most efficient and successful techniques due to its wide applications for fabrication of continuous fibers with diameters in a nanometer scale [18,19,20,21,22,23,24]. Electrospinning can be classified into different approaches such as needleless electrospinning, uniaxial electrospinning, coaxial electrospinning, and multifluidic electrospinning [24,25]. Uniaxial electrospinning is the most popular method for producing single-component nanofibers with tunable morphological and structural properties which may be applied in numerous applications of wearable electronic devices, tissue engineering, drug delivery, and wound healing [26,27,28,29,30]. The desired morphologies and structures of nanofibers can be achieved towards a diverse combination of complementary materials in the complicated core-shell composite structures fabricated via coaxial electrospinning [25,31,32,33,34]. Compared to uniaxial electrospinning in which a single needle is used, coaxial electrospinning requires two concentric needles with different polymer solutions to produce the core and shell of composite nanofibers [31,35,36]. Considerable efforts have been dedicated to the multifunctional properties and novelty of electrospun composite nanofibers which hold great potential for electronic devices, biomedicine, and tissue regeneration [35,37,38,39,40,41]. The superior combination of the complementary polymers in the core and shell structure is beneficial to the mechanical properties over the neat polymers [42,43]. The novel applications of coaxial electrospun core/shell composite nanofibers as scaffold materials have been explored towards their improved performances in proliferation, cell attachment, biocompatibility, and biodegradability [42,43,44].

In the present study, we utilized the coaxial electrospinning process to produce core/shell composite fibrous membranes, namely the core/shell-copolymer/terpolymer (core/shell-TrFE/CTFE) and core/shell terpolymer/copolymer (core/shell-CTFE/TrFE), in comparison with the pristine P(VDF-TrFE-CTFE) and P(VDF-TrFE) fabricated via the uniaxial electrospinning method. An intensive investigation of those four single and coaxial electrospun nanofibers was conducted in terms of their structural, mechanical, dielectric, and piezoelectric responses in order to evaluate the feasibility of coaxial composite fibrous membranes-based EAP actuators as artificial muscles.

## 2. Results

### 2.1. Nanofiber Morphology

**Figure 1:**
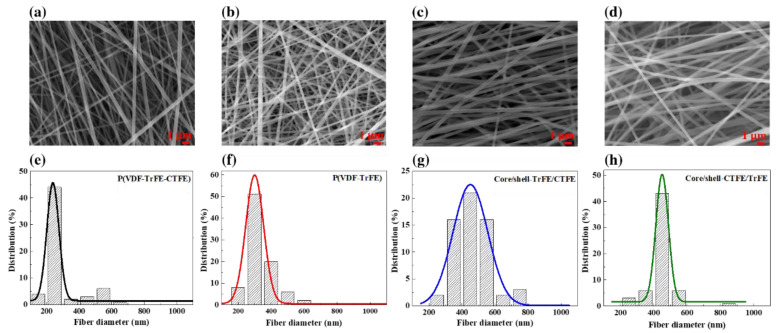
SEM images of the (**a**) pristine P(VDF-TrFE-CTFE), (**b**) pristine P(VDF-TrFE), (**c**) core/shell-TrFE/CTFE, and (**d**) core/shell-CTFE/TrFE nanofibers. The distribution of electrospun nanofibers in the (**e**) pristine P(VDF-TrFE-CTFE), (**f**) pristine P(VDF-TrFE), (**g**) core/shell-TrFE/CTFE, and (**h**) core/shell-CTFE/TrFE films.

Figure 1 depicts SEM micrographs of four different kinds of electrospun nanofiber films. The average nanofiber diameters of the pure P(VDF-TrFE-CTFE), pure P(VDF-TrFE), and coaxial electrospun core/shell composite sheets of CTFE/TrFE and TrFE/CTFE analyzed by imageJ [45] were determined to be 289, 283, 464, and 465 ± 5 nm, respectively. The average diameters of the coaxial electrospun composite nanofibers were approximately 1.6 times larger than those of the single electrospun nanofibers. The distribution of nanofibers in the core/shell-TrFE/CTFE was more uniform than that in the core/shell-CTFE/TrFE. The degree of nanofiber alignment in the single and coaxial fibrous membranes was determined as follows:

(1) $$S=2\cos^{2}\bar{\theta }-1$$

where *S* is the fiber orientation order parameter and $\bar{\theta }$ is the average angle between the fiber alignment and the preferred orientation direction [46,47]. The fibers are perfectly oriented when the value of *S* is 1, while they are randomly oriented when the value of *S* is 0. The larger the value of *S*, the higher the degree of fiber orientation.

The fiber orientation order parameters were characterized to be 0.46, 0.25, 0.61, and 0.47 in the P(VDF-TrFE-CTFE), P(VDF-TrFE), core/shell-TrFE/CTFE, and core/shell-CTFE/TrFE films, respectively. Among the four types of electrospun nanofibers, the neat P(VDF-TrFE) nanofibers were more randomly oriented, while the coaxial core/shell-TrFE/CTFE nanofibers were better aligned. It can be noted that there was a negligible discrepancy in density between the neat P(VDF-TrFE) and P(VDF-TrFE-CTFE), while the densities of the coaxial core/shell-TrFE/CTFE and core/shell-CTFE/TrFE were 2 and 1.5 times greater than those of the pure membranes. Such a denser arrangement of nanofibers in the coaxial core/shell structures was presumably assigned to strong interactions between the copolymer and terpolymer layers.

### 2.2. Mechanical Properties

**Figure 2:**
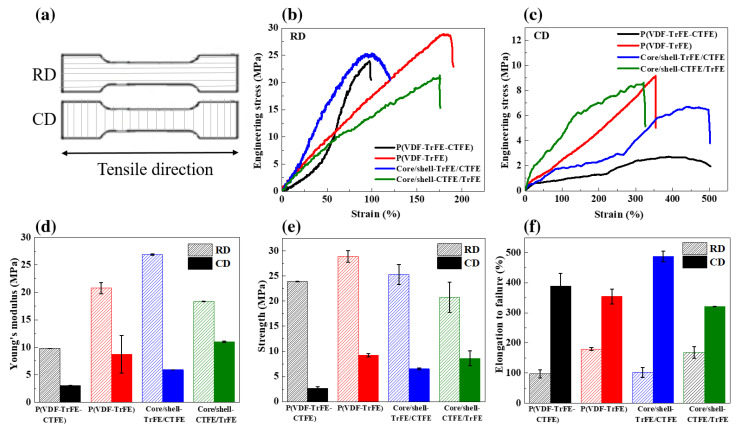
(**a**) Schematic illustration of tensile tests in the RD and CD. Engineering S-S curves of the four kinds of electrospun sheets in the (**b**) RD and (**c**) CD. (**d**) Young’s modulus, (**e**) tensile strength, and (**f**) elongation to failure of the single and coaxial electrospun nanofibers in both RD and CD.

Since the electrospun fibrous membranes exhibited an obvious degree of nanofiber orientation, the tensile tests were performed in both the revolving direction (RD) and cross direction (CD) to examine the response of aligned nanofibers to anisotropic mechanical properties [48]. The tensile direction was parallel and perpendicular to the oriented nanofibers in RD and CD, respectively, as illustrated in Figure 2a. Figure 2b,c describes the engineering stress-strain (S-S) curves in both RD and CD of the four types of electrospun films, respectively. The tensile strengths of the four membranes were higher in the RD, while their elongations at failure were larger in the CD, indicating an evident anisotropy of tensile properties between the RD and CD due to the highly aligned fibers along the RD.

The values of tensile modulus, tensile strength, and elongation determined from the S-S curves of the four eletrospun nanofiber sheets were presented in Figure 2d–f, respectively. A more pronounced mechanical performance of the copolymer P(VDF-TrFE) in both RD and CD was achieved compared with the terpolymer P(VDF-TrFE-CTFE). In the RD, the pure (PVDF-TrFE-CTFE) nanofibers owned an extremely low Young’s modulus of 9.8 MPa, tensile strength of 23.9 MPa, and poor elongation of 97.1% compared with a much higher Young’s modulus of 20.8 MPa, tensile strength of 28.9 MPa, and better elongation of 179.4% in the pure P(VDF-TrFE). Similarly, in the CD, the Young’s modulus of 8.7 MPa and tensile strength of 9.2 MPa in the pure P(VDF-TrFE) were much better than those of 3.1 and 2.7 MPa in the pure P(VDF-TrFE-CTFE). However, a relatively better elongation of 389.3% in the P(VDF-TrFE-CTFE) rather than that of 354.1% in the P(VDF-TrFE) was obtained. Such mechanical test results of the neat P(VDF-TrFE-CTFE) and P(VDF-TrFE) electrospun nanofibers were in accordance with previous studies [49,50].

The tensile properties of the core/shell composite nanofibers were successfully tailored by the coaxial electrospinning technique. The coaxial core/shell-CTFE/TrFE revealed the mechanical tunability between those of the neat P(VDF-TrFE-CTFE) and P(VDF-TrFE) nanofibers. Meanwhile, the coaxial core/shell-TrFE/CTFE membrane disclosed superior mechanical performances of higher Young’s modulus of 26.9 MPa and a comparable tensile strength of 25.3 MPa in the RD, accompanied with an excellent ductility of 487.2% with little sacrifice of strength in the CD compared with the single electrospun fibrous membranes. Our results were in agreement with the previous reports that the core/shell composite nanofibers fabricated using the coaxial electrospinning process exhibited more salient mechanical properties over the neat materials [42,43]. It can be noted that the coaxial core/shell-TrFE/CTFE showed a more striking anisotropy in tensile properties between the RD and CD, due to a stronger preferred orientation of nanofibers along the CD. The coaxial electrospun nanofibers may be potentially applied as implantable artificial muscles since their tensile moduli are more appropriate to those of natural mammalian skeletal muscles (10–60 MPa) [51].

The mechanical response of the coaxial electrospun nanofibers is strongly governed by combined effects of the polymer material types in the core and shell, morphology and diameter distribution of nanofibers, degree of nanofiber alignment, density of electrospun membrane, chemical and physical interactions of the polymer chains, and defects or impurities [42]. A higher degree of nanofiber alignment coupled with a tightly packed membrane gave rise to the significant enhancement in the tensile properties of the coaxial core/shell-TrFE/CTFE structure.

### 2.3. Tensile Moduli of the Coaxial Electrospun Core/Shell Composite Nanofibers

To study how the volume fraction of fibers affects the tensile moduli of the coaxial electrospun composite structures, the experimentally measured tensile moduli of the core/shell composite nanofibers were compared with the theoretically predicted moduli calculated from the two individual polymer components using the upper bound (Equation (2)) and the lower bound (Equation (3)) for the rule of mixtures [52]. The lower bound represents the transverse loading direction in which the fibers do not reinforce the matrix [52].

(2) $$E_{C}=E_{m}\times V_{m}+E_{f}\times V_{f}$$

(3) $$E_{C}=\frac{E_{m}E_{f}}{V_{m}E_{f}+V_{f}E_{m}}$$

where *E_c_* is the modulus of the composite, *E_m_* and *E_f_* are the moduli of the matrix and fibers, *V_m_* and *V_f_* are the volume fraction of the matrix and fibers, respectively.

**Figure 3:**
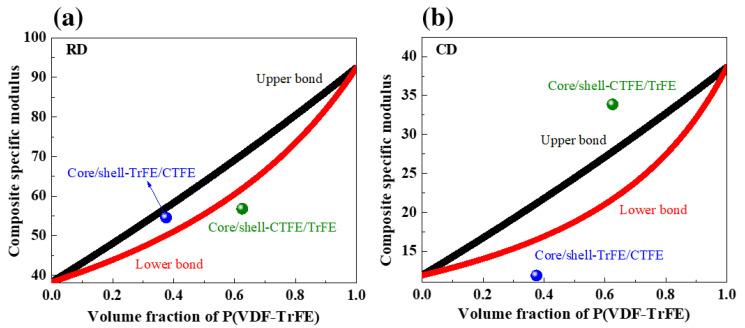
The upper and lower bounds calculated by the rule of mixtures accompanied with the experimentally measured specific Young’s moduli of the coaxial electrospun composite nanofibers in the (**a**) RD and (**b**) CD.

Figure 3 illustrates the calculated upper and lower bounds for the rule of mixtures as a function of the volume fraction of copolymer P(VDF-TrFE) accompanied with the experimentally measured specific Young’s moduli of the coaxial composite films [52]. In the RD, the experimentally measured tensile moduli of the coaxial composite nanofibers slightly increased with the increasing volume fraction of P(VDF-TrFE) within the range between the theoretically calculated upper and lower bounds and quite fitted with the rule of mixtures. However, in the CD, with the increasing volume fraction of P(VDF-TrFE) in the shell, the experimentally measured tensile moduli of the coaxial composite nanofibers were beyond the theoretically calculated upper and lower bounds. Such a considerable difference in tensile moduli between the two measured coaxial composite sheets was ascribed to their remarkably anisotropic orientation.

### 2.4. Structural Characterization

**Figure 4:**
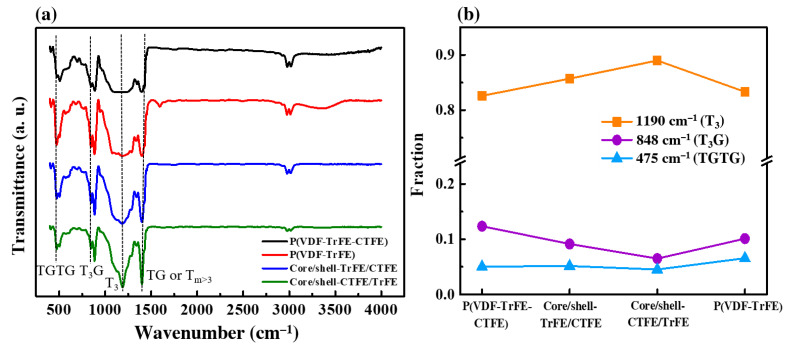
(**a**) FTIR spectra and (**b**) fraction of each chain conformation in the single and coaxial electrospun nanofibers.

To better understand the role of other possible reasons contributing to the mechanical response of four electrospun membranes, Fourier-transform infrared spectroscopy (FTIR) was conducted to investigate the chain conformation in the single and coaxial electrospun films. Figure 4a depicts the transmission spectra of the four electrospun membranes which exhibited three major different kinds of chain conformational structures, namely, T_3_ (1190 cm^−1^), T_3_G (848 cm^−1^), and TGTG (475 cm^−1^) [10,53]. No evident peak located at 1190 cm^−1^ was visible in the single electrospun nanofibers, however, that peak was significantly sharper in the coaxial composite structures, representing the C-F bond in the T_3_ conformation. Since the bonding energy of the C-F bond is stronger than that of the other bonds, the increased C-F bond was presumably ascribed to the enhancement in Young’s modulus and tensile strength of the coaxial electrospun films.

In order to identify the variation in the reflection intensities of the three major chain conformations (all–trans T_3_, trans*–*gauche T_3_G, and short trans TGTG) in the single and coaxial electrospun films [10,53], the fraction of each conformation was calculated using Equation (4).

(4) $$F_{i}=\frac{A_{i}}{A_{1}+A_{2}+A_{3}}$$

where *i* = 1, 2, 3, and *A*_1_, *A*_2_, and *A*_3_ are the integrated areas of T_3_, T_3_G, and TGTG chain conformations, respectively.

Figure 4b describes the fraction of each chain confirmation in the single and coaxial electrospun nanofibers. At a low copolymer P(VDF-TrFE) volume portion of 37.5% in the core, there was an increase of all*–*trans T_3_ and a decrease of short trans TTTG conformation in the core/shell-TrFE/CTFE compared with the neat P(VDF-TrFE-CTFE) and P(VDF-TrFE) sheets. With the increase of copolymer volume portion of 62.5% in the shell, the ratio of highly polar ferroelectric T_3_ reached the highest value, while that of polar relaxor ferroelectric T_3_G dropped to the lowest value, implying a more pronounced impact of copolymer-based shell in the enhanced FE phase accompanied with the reduced RFE phase in the coaxial core/shell-CTFE/TrFE composite structure. A negligible variation of nonpolar TGTG conformation was seen in the single and coaxial nanofibers.

**Figure 5:**
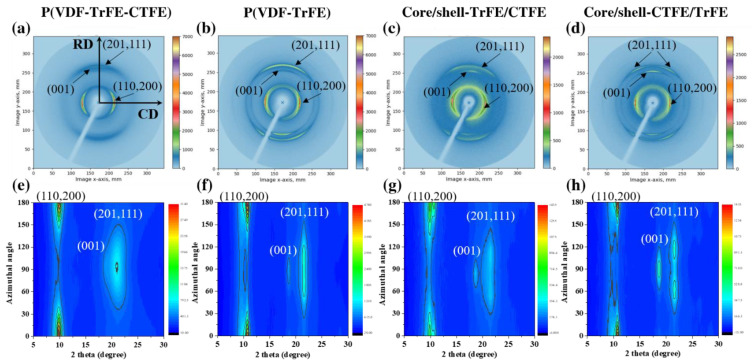
The 2D WAXD patterns in the (**a**) P(VDF-TrFE-CTFE), (**b**) P(VDF-TrFE), (**c**) core/shell-TrFE/CTFE, and (**d**) core/shell-CTFE/TrFE. Azimuthal profiles as a function of the 2θ angle in the (**e**) P(VDF-TrFE-CTFE), (**f**) P(VDF-TrFE), (**g**) core/shell-TrFE/CTFE, and (**h**) core/shell-CTFE/TrFE.

Since the electrospun films showed an anisotropic orientation of nanofibers, the wide-angle X-ray diffraction (WAXD) was employed to explore the preferred crystallographic texture orientation in the single/coaxial electrospun fibrous membranes. Figure 5 presents two-dimensional (2D) WAXD patterns and corresponding azimuthally integrated 2D WAXD patterns as a function of the 2θ angle in the four electrospun nanofibers. More details of the related protocols are archived [49,54,55]. There was an obvious orientation anisotropy in which a preferred (110,200)_β_ reflection in the crystalline β phase along the CD and preferred (001)_β_ and (201,111)_β_ crystallographic planes along the RD were obtained in the four electrospun sheets [12,14]. The pristine terpolymer P(VDF-TrFE-CTFE) revealed partially superimposed reflections of (001)_β_ and (201,111)_β_, while the copolymer P(VDF-TrFE) disclosed their distinct reflections.

**Figure 6:**
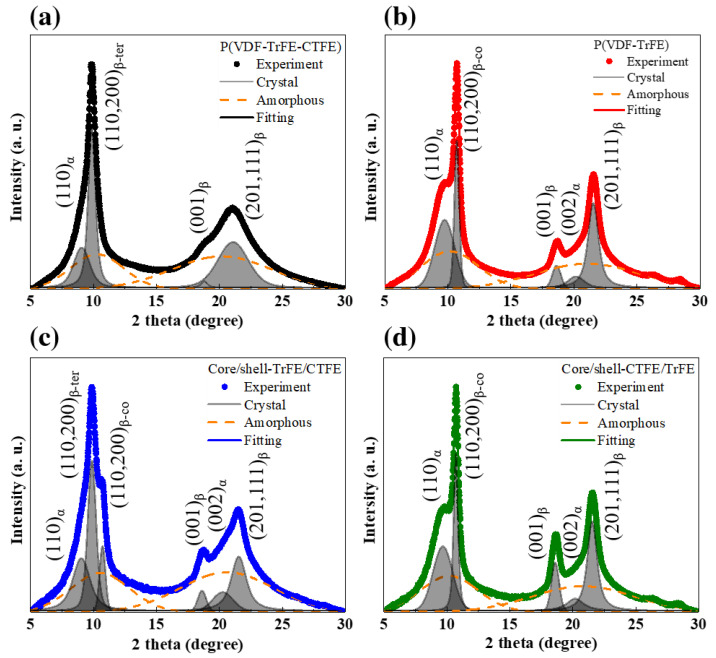
The 1D WAXD intensity profiles and corresponding peak fitting in the (**a**) pristine P(VDF-TrFE-CTFE), (**b**) pristine P(VDF-TrFE), (**c**) core/shell-TrFE/CTFE, and (**d**) core/shell-CTFE/TrFE.

To acquire a further analysis in the evolution of crystallographic planes, the corresponding one-dimensional (1D) intensity profiles of the four electrospun sheets were shown in Figure 6. The diffraction profiles were deconvoluted with a Voigt function using the Fityk software [56] and Bragg reflections were indexed following the previously reported works [9,12]. The (110,200)_β_ reflection in the RFE crystalline P(VDF-TrFE-CTFE) was located at 2θ = 9.9° in Figure 6a and that in the highly polar FE crystalline P(VDF-TrFE) was situated at 2θ = 10.7° in Figure 6b. In Figure 6c, the core/shell-TrFE/CTFE exhibited a coexistence of both (110,200)_β_ reflections from the RFE and FE crystallines, implying a partial infiltration of copolymer with terpolymer chains. On the other hand, only the (110,200)_β_ reflection in the FE crystalline β phase from the copolymer was visible in the coaxial core/shell-CTFE/TrFE sheet, as shown in Figure 6d, which infers a complete infiltration of the copolymer with terpolymer chains and their cocrystallization phenomenon.

To clarify the effect of coaxial electrospinning on the crystalline properties of the composite nanofibers, the degree of crystallinity and polar crystalline domain size were extensively examined. The degree of crystallinity (*X*_c_) was defined according to the following equation:

(5) $$X_{c}=A_{c}/(A_{c}+A_{a})$$

where *A*_c_ and *A*_a_ are the integrated areas of the crystalline peaks and amorphous regions, respectively [12].

The degree of crystallinity was determined to be 44.5, 50.9, 45.6, and 56.7% in the P(VDF-TrFE-CTFE), P(VDF-TrFE), core/shell-TrFE/CTFE, and core/shell-CTFE/TrFE, respectively. The pristine P(VDF-TrFE) showed better crystallinity than the pristine P(VDF-TrFE-CTFE). A higher degree of crystallinity in the coaxial composite nanofibers rather than in the pristine terpolymer was obtained.

The crystallite size (*L*) representing the RFE or FE domain size of the corresponding (110,200)_β_ crystallographic plane was calculated using the Scherrer equation:

(6) L=0.9λ/Bcosθ

where *λ* is the X-ray wavelength, *B* is the full width at half-maximum (FWHM), and *θ* is the angle of the diffraction peak.

The crystallite sizes of the (110,200)_β_ reflection in the single and coaxial electrospun nanofibers were listed in Table 1. The crystallite size of (110,200)_β-ter_ in the pristine terpolymer was 14.1 nm, which was smaller than 18.2 nm of (110,200)_β-co_ in the pristine copolymer. The addition of the third monomer CTFE considered as a defect causes the break of a larger micrometer-sized FE into smaller nanometer-sized RFE crystal domains, thus decreasing the FE crystalline domain size and crystallinity of the copolymer P(VDF-TrFE) [11,57]. The core/shell-TrFE/CTFE included both RFE and RE crystalline sizes of 13.6 and 14.9 nm, which were smaller than those in the pristine terpolymer of 14.1 nm and copolymer of 18.2 nm, respectively. The cocrystallization that occurred at a higher volume fraction of copolymer P(VDF-TrFE) in the shell facilitates the growth of only the coherent polar FE crystalline and hence, resulted in the largest FE crystallite domain size of 19.7 nm in the core/shell-CTFE/TrFE. The favorable orientation of a larger FE crystalline domain size towards the CD was presumably responsible for the measured tensile modulus beyond the calculated upper bond observed in the core/shell-CTFE/TrFE structure in Figure 3b.

### 2.5. Dielectric Response

**Figure 7:**
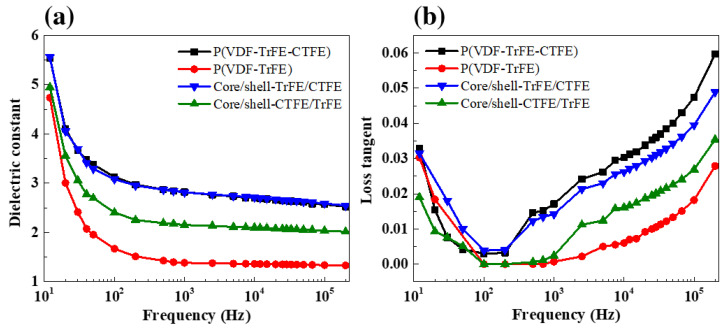
(**a**) Dielectric constant and (**b**) dielectric loss as a function of frequency in the single and coaxial electrospun nanofibers.

Figure 7 presents the dielectric response as a function of frequency at 1 V in the single and coaxial electrospun films. In Figure 7a, all single and coaxial electrospun sheets had the highest dielectric constants at the lowest frequency of 12 Hz, which was attributed to the Maxwell-Wagner-Sillars (MWS) interfacial polarization and space charge effects [32,58,59,60]. The dielectric constants drastically decreased with a slight increase of low frequency from 12 to 20 Hz since it was too difficult for the molecular dipoles to quickly align themselves with respect to a rapid alternation of the applied electric field direction [32,61]. The slightly decreasing trend in the dielectric constant response at subsequent higher frequencies was derived from the dipolar polarization inside the electrospun nanofibers. A similar decreasing tendency in the dielectric loss was observed within the low frequency region from 12 to 100 Hz, as shown in Figure 7b. The dielectric loss then increased with the increasing frequency above 103 Hz due to the polarization loss and DC conduction loss [61,62].

The dielectric constant of the pure P(VDF-TrFE-CTFE) was obviously higher than that of the pure P(VDF-TrFE) over the whole frequency range due to the nanopolar regions of RFE in the terpolymer [9,10,63]. The dielectric constants of the pure P(VDF-TrFE-CTFE), P(VDF-TrFE), core/shell-TrFE/CTFE, and core/shell-CTFE/TrFE at 12 Hz were found to be 5.5, 4.7, 5.5, and 4.9, respectively. Higher dielectric constants in both coaxial composite structures rather than in the pure copolymer were reasonably obtained. It is noted that the dielectric constant in the core/shell-TrFE/CTFE was similar to that in the pure terpolymer, which originated from the induced transformation from FE to RFE via interfacial couplings coupled with the reduced RFE crystalline domain size at a smaller volume fraction of the copolymer in the core. Meanwhile, a larger volume fraction of the copolymer in the shell reduced the dielectric constant in the core/shell-CTFE/TrFE compared with that in the pristine terpolymer due to a more predominant effect of FE crystalline domains in the copolymer.

## 3. Discussion

**Figure 8:**
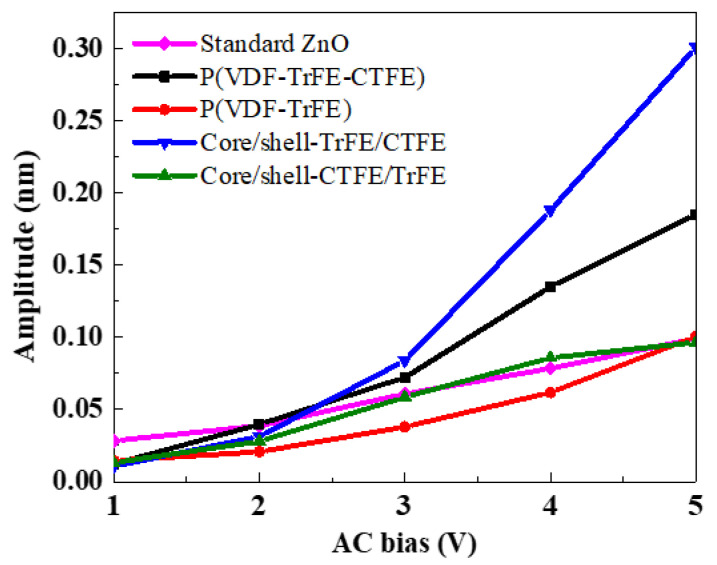
Piezoelectric signal versus the applied AC voltage in the single and coaxial electrospun nanofibers.

The dielectric response under a low electric field is not sufficient enough to reflect the overall polarization mechanism of electrospun nanofibers, therefore, it is of great need to measure the piezoelectric response under a high electric field. The local piezoelectric performance was conducted using piezoresponse force microscopy (PFM) [64]. The individual electrospun nanofiber was polarized at 10 V under a carbon probe. The piezoelectric amplitude response can be obtained by applying an AC voltage with a frequency of 1 kHz and a voltage range from 1 to 5 V, as shown in Figure 8.

The slope of piezoelectric amplitude response in the four electrospun nanofibers represents the effective piezoelectric constant (d_33,eff_), which was determined by comparison with the slope of the standard ZnO sample.

The slope and corresponding d_33,eff_ in the single and coaxial electrospun films were listed in Table 2. The d_33,eff_ of the pure P(VDF-TrFE-CTFE) was 30.2 pm/V, twice higher than that of the pure P(VDF-TrFE) (14.6 pm/V), presumably attributed to the higher saturation polarization under a high electric field of the terpolymer [65]. The d_33,eff_ of the coaxial core/shell-CTFE/TrFE (15.4 pm/V) was slightly higher than that of the copolymer, while the d_33,eff_ of the core/shell-TrFE/CTFE reached the highest value of 50.5 pm/V, due to the coexistence of RFE and FE crystalline domains associated with highly aligned dipole moments. Such a very outstanding d_33,eff_ in the coaxial core/shell-TrFE/CTFE was very close to the previously reported d_33,eff_ value of the annealed terpolymer (52 pC/N) [66]. Therefore, better piezoelectric properties are expectedly achieved through additional annealing treatments of the coaxial electrospun composite nanofibers.

In general, the coaxial electrospun core/shell-TrFE/CTFE composite nanofiber membrane manifested superior mechanical properties over the pristine polymers, which was similarly obtained in other electrospun composite nanofibers [42,43]. Furthermore, the remarkably improved dielectric and piezoelectric properties together with the enhanced Young’s modulus in the core/shell-TrFE/CTFE composite structure enabled potentially promising applications of EAP actuators as artificial muscles.

## 4. Materials and Methods

### 4.1. Sample Preparation

The copolymer P(VDF-TrFE) (75/25 mol%) and terpolymer P(VDF-TrFE-CTFE) (61.7/30.4/7.9 mol%) powders were from Arkema Group (PiezoTech, France). The polymer powder was dissolved in a cosolvent of DMAc (ACROS Organics, Geel, Belgium) and MEK (ACROS Organics, USA) with a ratio of 2:3, the mixture was then heated at 60 °C for 3–4 h using a magnetic stirring. The solutions of 13 wt% P(VDF-TrFE) and 13 wt% P(VDF-TrFE-CTFE) were obtained and cooled to room temperature. Four different kinds of electrospun nanofibers, namely, pristine P(VDF-TrFE), pristine P(VDF-TrFE-CTFE), and coaxial core/shell composite structures of CTFE/TrFE and TrFE/CTFE nanofibers were fabricated. The neat P(VDF-TrFE) and P(VDF-TrFE-CTFE) films were fabricated using uniaxial electrospinning, while the two various core/shell structures were produced using the coaxial electrospinning technique.

### 4.2. Electrospinning

The uniaxial and coaxial electrospinning processes were performed at 24 °C with a relative humidity of 55% using a self-made electrospinning machine. A 10 mL syringe pump and a 20 G stainless steel needle (inner diameter of 0.6 mm) filled with the blend solution were driven at a feeding rate of 1 mL/h in the uniaxial electrospinning process.

The coaxial electrospinning process requires two concentrically detachable stainless steel needles, 20 G as outer and 26 G (inner diameter of 0.26 mm) as inner ones. The schematic illustration of coaxial electrospinning can be found elsewhere [24,25,31,36]. The P(VDF-TrFE) and P(VDF-TrFE-CTFE) solutions were injected in two separate 10 mL syringe pumps and driven at the feeding rates of 0.6 and 1 mL/h for the core and shell, respectively. The volume fraction of the core/shell polymers was 0.375:0.625.

The collector was a custom-made rotating cylindrical drum with a diameter of 18 cm and rotated at a speed of 800 rpm. A high DC voltage of 18 kV was applied between the positive electrode (connected to the needle) and negative electrode (connected to the collector) at a working distance of 18 cm.

### 4.3. Structural Characterization

Electrospun nanofiber morphology was analyzed using the scanning electron microscope (SEM, JEOL JSM-6700F) operating at 15 KeV. The samples for SEM measurements were sputter-coated with platinum. The chain conformation in single and coaxial electrospun nanofibers was measured by Fourier-transform infrared spectroscopy (FTIR) using a Perkin Elmer spectrum 100 spectrometer in the frequency range of 400–4000 cm^−1^.

### 4.4. Tensile Test

The four electrospun membranes with the thickness in the range of 20–125 μm measured by the cross-sectional SEM were cut into dog-bone shaped specimens with a total length of 50 mm, a gauge length and width of 10 and 3 mm, respectively. The tensile test was conducted by a micro-stretching machine equipped with a 50 kg load cell at a stretch rate of 0.1 mm/min until failure. The specimens were strained in both the RD and CD, parallel and perpendicular to the alignment of nanofibers, respectively.

### 4.5. Wide-Angle X-ray Diffraction

The non-destructive wide-angle X-ray diffraction was implemented at the beamline (BL) 01C2, National Synchrotron Radiation Research Center (NSRRC), Taiwan. Diffraction patterns were recorded by a 2D detector to acquire the preferred orientation of crystallographic planes.

### 4.6. Electrical Properties

The dielectric response as a function of frequency from 12 to 200 kHz was measured using a LCR-821 impedance analyzer at 1 V at room temperature. The effective piezoelectric constant was acquired using the PFM function in the Park XE7 atomic force microscope (AFM) coupled with a phase-locked amplifier.

## 5. Conclusions

The effective tunability of structural, mechanical, dielectric, and piezoelectric properties of the core/shell composite nanofibers consisting of terpolymer P(VDF-TrFE-CTFE) and copolymer P(VDF-TrFE) was successfully attained via the coaxial electrospinning approach. The coaxial electrospun core/shell films revealed a better alignment and denser packing of nanofibers accompanied with stronger C-F bonding compared with the neat polymers. Such outstanding combined effects contributed to the significant enhancement in Young’s modulus coupled with a superior tensile strength and elongation to rupture, especially achieved in the core/shell-TrFE/CTFE. The coaxial core/shell-TrFE/CTFE composite nanofiber membrane exhibited more pronounced mechanical, dielectric, and piezoelectric performances, which enables potentially promising applications of EAP actuators as artificial muscles.

## Figures and Tables

**Table 1:** The 2θ, lattice spacing, and crystallite size of the (110,200)_β_ reflection in the single and coaxial electrospun nanofibers.

Sample	(110,200)_β-ter_			(110,200)_β-co_		
	2θ (°)	d (Å)	L (nm)	2θ (°)	d (Å)	L (nm)
P(VDF-TrFE-CTFE)	9.9	4.795	14.1			
P(VDF-TrFE)				10.7	4.420	18.2
Core/shell-TrFE/CTFE	9.9	4.785	13.6	10.7	4.433	14.9
Core/shell-CTFE/TrFE				10.7	4.420	19.7

**Table 2:** The slope of piezoelectric amplitude response and corresponding d_33,eff_ in the four electrospun nanofibers.

Sample	Slope	d_33,eff_ (pm/V)
Standard ZnO	0.6036	12.4
P(VDF-TrFE-CTFE)	1.4691	30.2
P(VDF-TrFE)	0.7099	14.6
Core/shell-TrFE/CTFE	2.4564	50.5
Core/shell-CTFE/TrFE	0.7474	15.4

## Abbreviations

EAPs	Electroactive polymers
P(VDF-TrFE)	Poly(vinylidene fluoride-trifluoroethylene)
CTFE	Chlorotrifluoroethylene
P(VDF-TrFE-CTFE)	Poly(vinylidene fluoride-trifluoroethylene-chlorotrifluoroethylene)
RFE	Relaxor ferroelectric
FE	Ferroelectric
RD	Revolving direction
CD	Cross direction
S-S	Stress–strain
FTIR	Fourier-transform infrared spectroscopy
WAXD	Wide-angle X-ray diffraction
2D	Two-dimensional
1D	One-dimensional
MWS	Maxwell-Wagner-Sillars
PFM	Piezoresponse force microscopy
SEM	Scanning electron microscope
NSRRC	National Synchrotron Radiation Research Center
AFM	Atomic force microscope
